# Suicide Trends Among and Within Urbanization Levels by Sex, Race/Ethnicity, Age Group, and Mechanism of Death — United States, 2001–2015

**DOI:** 10.15585/mmwr.ss6618a1

**Published:** 2017-10-06

**Authors:** Asha Z. Ivey-Stephenson, Alex E. Crosby, Shane P. D. Jack, Tadesse Haileyesus, Marcie-jo Kresnow-Sedacca

**Affiliations:** 1 Division of Violence Prevention, National Center for Injury Prevention and Control, CDC; 2 Division of Analysis, Research, and Practice Integration, National Center for Injury Prevention and Control, CDC

## Abstract

**Problem/Condition:**

Suicide is a public health problem and one of the top 10 leading causes of death in the United States. Substantial geographic variations in suicide rates exist, with suicides in rural areas occurring at much higher rates than those occurring in more urban areas. Understanding demographic trends and mechanisms of death among and within urbanization levels is important to developing and targeting future prevention efforts.

**Reporting Period:**

2001–2015.

**Description of System:**

Mortality data from the National Vital Statistics System (NVSS) include demographic, geographic, and cause of death information derived from death certificates filed in the 50 states and the District of Columbia. NVSS was used to identify suicide deaths, defined by *International Classification of Diseases, 10th Revision* (ICD-10) underlying cause of death codes X60–X84, Y87.0, and U03. This report examines annual county level trends in suicide rates during 2001–2015 among and within urbanization levels by select demographics and mechanisms of death. Counties were collapsed into three urbanization levels using the 2006 National Center for Health Statistics classification scheme.

**Results:**

Suicide rates increased across the three urbanization levels, with higher rates in nonmetropolitan/rural counties than in medium/small or large metropolitan counties. Each urbanization level experienced substantial annual rate changes at different times during the study period. Across urbanization levels, suicide rates were consistently highest for men and non-Hispanic American Indian/Alaska Natives compared with rates for women and other racial/ethnic groups; however, rates were highest for non-Hispanic whites in more metropolitan counties. Trends indicate that suicide rates for non-Hispanic blacks were lowest in nonmetropolitan/rural counties and highest in more urban counties. Increases in suicide rates occurred for all age groups across urbanization levels, with the highest rates for persons aged 35–64 years. For mechanism of death, greater increases in rates of suicide by firearms and hanging/suffocation occurred across all urbanization levels; rates of suicide by firearms in nonmetropolitan/rural counties were almost two times that of rates in larger metropolitan counties.

**Interpretation:**

Suicide rates in nonmetropolitan/rural counties are consistently higher than suicide rates in metropolitan counties. These trends also are observed by sex, race/ethnicity, age group, and mechanism of death.

**Public Health Action:**

Interventions to prevent suicides should be ongoing, particularly in rural areas. Comprehensive suicide prevention efforts might include leveraging protective factors and providing innovative prevention strategies that increase access to health care and mental health care in rural communities. In addition, distribution of socioeconomic factors varies in different communities and needs to be better understood in the context of suicide prevention.

## Introduction

Suicides, the fatal component of suicidal behavior, result from an interaction of individual, interpersonal, social, and environmental influences (*1*). These deaths take a toll on society. In 2015, suicide ranked as the 10th leading cause of death in the United States and was the cause of 44,193 deaths (*2*). One of the *Healthy People 2020* objectives is to reduce suicides by 10% (from 11.3 suicides per 100,000 population in 2007 to 10.2 by 2020); however, during 2005–2015 the age-adjusted suicide rate increased 21.6% whereas many other causes of mortality have declined (*3**,**4*). The increase has been manifested across multiple demographic groups (*3*). One study, after adjusting for underreporting, estimated the 2013 U.S. national cost of suicide and suicide attempts at $93.5 billion (*5*).

Patterns of suicide have been associated with various sociodemographic characteristics. For example, in the United States, suicide rates are higher for males than for females and for adults aged ≥45 years than for adolescents and young adults; overall rates are higher for non-Hispanic white and American Indian/Alaska Native populations than for other racial/ethnic groups (*6*). Recent trends have demonstrated an increase in suicides, particularly among working age adults aged 45–64 years (*3*). Previous studies have documented regional and state differences in suicide rates in the United States (*7**–**10*). Suicide rates tend to be higher in the West than in the South, Midwest, and Northeast. In at least one study, regional differences in demographic patterns (i.e., age, race/Hispanic ethnicity, and sex) did not account for variations in suicide rates (*7*), indicating that some other factor, possibly population density (*11*), might be influencing the differences. An analysis of urban-rural differences focused on specific age groups (e.g., youths and older adults) and highlighted the disparities (*8**,**9*). In addition, the level of urbanization is associated with suicide rates. One study analyzed death certificate data during 1999–2015 and reported that suicide rates in less urban areas are higher than in more urban areas and the gap in rates has been widening (*10*). This report analyzes death certificate data during 2001–2015 to describe patterns of suicide by level of urbanization, demographic characteristics, and mechanism of death in the United States. Public health professionals and prevention program staff can use the data to identify specific populations in need of targeted interventions to address suicide rates.

## Methods

The National Vital Statistics System annual compressed mortality data files during 2001–2015 were used to identify suicides using *International Classification of Diseases, 10th Revision* (ICD-10) underlying cause of death codes X60–X84, Y87.0, and U03, excluding foreign residents (*12*). Three-year moving averages were used to reduce the variability of a small number of observations in a particular period. Working backward from 2015 (the most recent data year), 2001 was identified as the starting point. Going back further would have required the inclusion of 1998 data, which use the *International Classification of Diseases, 9th Revision* (ICD-9) for coding cause of death, rather than ICD-10 codes. Children aged <10 years (74 deaths) were excluded because intent for self-harm is not usually attributed to young children. Annual suicide counts were summarized using the 2006 National Center for Health Statistics urban-rural classification scheme for counties (*13*). The six-level 2006 classification scheme was selected to coincide with the middle of the study period and to align with the results of a recent report on trends by level of urbanization (*2**,**10**,**12*). Following are the six classification levels for counties:

1. Large central metropolitan is part of a metropolitan statistical area with ≥1 million population and includes a principal city.
2. Large fringe metropolitan is part of a metropolitan statistical area with ≥1 million population but does not include a principal city.
3. Medium metropolitan is part of a metropolitan statistical area with ≥250,000 but <1 million population.
4. Small metropolitan is part of a metropolitan statistical area with <250,000 population.
5. Micropolitan (nonmetropolitan) is part of a micropolitan statistical area (has an urban cluster of ≥10,000 but <50,000 population).
6. Noncore (nonmetropolitan) is not part of a metropolitan or micropolitan statistical area.

Levels of urbanization were further collapsed into large metropolitan, medium/small metropolitan, and nonmetropolitan/rural for selected analyses. Large metropolitan includes counties from large central metropolitan and large fringe metropolitan areas. Medium/small metropolitan includes counties from medium and small metropolitan areas. Nonmetropolitan/rural includes counties from micropolitan and noncore areas.

U.S. Census Bureau county level population estimates were used to calculate annual suicide rates (per 100,000 population among persons aged ≥10 years). Rates were age adjusted to the 2000 U.S. standard population (*3**,**10*). Joinpoint regression analyses were used to examine the magnitude and direction of the annual trends in suicide rates for each level of urbanization. During the study period, there were two joinpoints in the large metropolitan counties and one joinpoint in all other levels of urbanization at p value <0.05 statistical significance level. Data were analyzed using SAS version 9.4 (SAS Institute, Inc., Cary, North Carolina) and Joinpoint version 4.4.0 (Statistical Methodology and Applications Branch, Surveillance Research Program, National Cancer Institute, Bethesda, Maryland) software.

## Results

**FIGURE 1:**
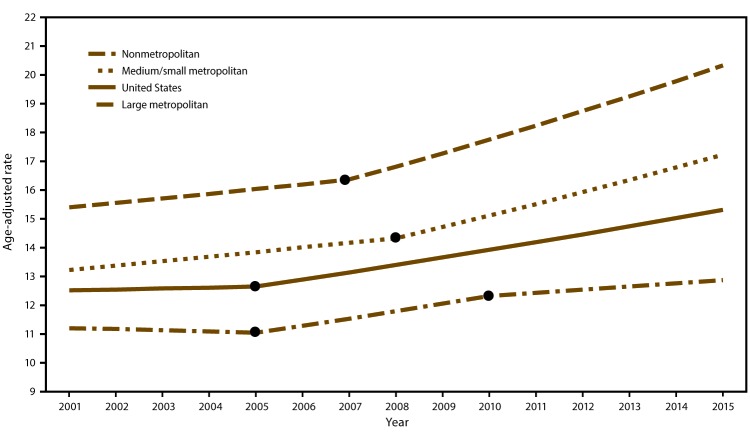
Suicide rates* among persons aged ≥10 years, by county urbanization level^†^ — United States, 2001–2015^§^ * Per 100,000 residents aged ≥10 years, age adjusted to the 2000 U.S. standard population. ^†^ Levels of urbanization were collapsed using the 2006 National Center for Health Statistics urban classification scheme. The six classification levels for counties are 1) large central metropolitan (part of a metropolitan statistical area with ≥1 million population and includes a principal city); 2) large fringe metropolitan (part of a metropolitan statistical area with ≥1 million population but does not include a principal city); 3) medium metropolitan (part of a metropolitan statistical area with ≥250,000 but <1 million population); 4) small metropolitan (part of a metropolitan statistical area with <250,000 population); 5) micropolitan (nonmetropolitan) (part of a micropolitan statistical area [has an urban cluster of ≥10,000 but <50,000 population]); and 6) noncore (nonmetropolitan) (not part of a metropolitan or micropolitan statistical area). Large metropolitan includes counties from large central metropolitan and large fringe metropolitan areas. Medium/small metropolitan includes counties from medium metropolitan and small metropolitan areas. Nonmetropolitan includes counties from micropolitan and noncore areas. ^§ ^Joinpoint regression analysis was used to determine annual percentage change with statistically significant trend (p<0.05). Dots indicate the joinpoints.

During 2001–2015, a total of 256,511 suicides were reported in large metropolitan, 173,045 in medium/small metropolitan, and 114,559 in nonmetropolitan/rural counties (Table). Joinpoint regression analyses indicated increases in annual age-adjusted suicide rates across the three urbanization levels during the study period, with counties in more urban areas having the lowest suicide rates and counties in less urban areas having the highest suicide rates (Figure 1). In large metropolitan counties, annual suicide rates decreased during 2001–2005, then increased during 2005–2010; these changes in rates were significant. During 2010–2015, although suicide rates continued to increase in large metropolitan counties, these increases were not significant. In nonmetropolitan/rural and medium/small metropolitan counties, increases in suicide rates occurred during 2001–2007 and the increases accelerated in 2007 and 2008. 

**TABLE:** Suicide deaths and rates* among persons aged ≥10 years, by county urbanization level,^†^ sex, race/ethnicity, age group, and mechanism of death — United States, 2001–2015

Characteristic	2001‒2003		2004‒2006		2007‒2009		2010‒2012		2013‒2015		Total	
	Deaths	Rates	Deaths	Rates	Deaths	Rates	Deaths	Rates	Deaths	Rates	Deaths	Rates
**County urbanization level**												
Large metropolitan	44,568	11.19	45,934	11.11	50,914	11.84	55,852	12.47	59,243	12.72	**256,511**	**11.92**
Medium/small metropolitan	28,936	13.39	31,095	13.82	33,892	14.48	37,539	15.54	41,583	16.77	**173,045**	**14.86**
Nonmetropolitan/rural	19,832	15.50	21,006	16.10	22,385	16.79	24,626	18.35	26,710	19.74	**114,559**	**17.32**
**Total**	**93,336**	**12.54**	**98,035**	**12.72**	**107,191**	**13.41**	**118,017**	**14.29**	**127,536**	**14.98**	**544,115**	**13.64**
**Sex**												
Female												
Large metropolitan	9,494	4.59	10,277	4.79	11,388	5.11	12,643	5.47	14,140	5.91	**57,942**	**5.20**
Medium/small metropolitan	5,626	5.09	6,455	5.63	7,159	6.02	8,084	6.58	9,411	7.51	**36,735**	**6.20**
Nonmetropolitan/rural	3,297	5.20	3,797	5.87	4,116	6.28	4,602	7.05	5,281	8.06	**21,093**	**6.50**
**Total**	**18,417**	**4.82**	**20,529**	**5.19**	**22,663**	**5.54**	**25,329**	**6.02**	**28,832**	**6.68**	**115,770**	**5.68**
Male												
Large metropolitan	35,074	18.69	35,657	18.20	39,526	19.30	43,209	20.19	45,103	20.20	**198,569**	**19.38**
Medium/small metropolitan	23,310	22.61	24,640	22.84	26,733	23.67	29,455	25.18	32,172	26.68	**136,310**	**24.28**
Nonmetropolitan/rural	16,535	26.47	17,209	26.84	18,269	27.68	20,024	29.90	21,429	31.62	**93,466**	**28.54**
**Total**	**74,919**	**21.17**	**77,506**	**21.02**	**84,528**	**21.99**	**92,688**	**23.23**	**98,704**	**23.92**	**428,345**	**22.34**
**Race/Ethnicity**												
White (NH)												
Large metropolitan	35,459	13.73	36,251	13.95	40,407	15.38	44,009	16.58	46,252	17.24	**202,378**	**15.40**
Medium/small metropolitan	25,425	15.21	27,057	15.87	29,373	16.91	32,570	18.53	35,808	20.24	**150,233**	**17.37**
Nonmetropolitan/rural	18,033	16.76	18,941	17.40	20,196	18.34	22,173	20.19	24,041	21.96	**103,384**	**18.92**
**Total**	**78,917**	**14.79**	**82,249**	**15.23**	**89,976**	**16.44**	**98,752**	**17.89**	**106,101**	**19.11**	**455,995**	**16.71**
Black (NH)												
Large metropolitan	3,750	6.49	3,712	6.17	3,819	6.11	4,240	6.50	4,491	6.56	**20,012**	**6.36**
Medium/small metropolitan	1,386	6.43	1,461	6.34	1,524	6.29	1,663	6.50	1,840	6.89	**7,874**	**6.49**
Nonmetropolitan/rural	578	5.48	656	6.18	660	5.93	677	6.12	695	6.07	**3,266**	**5.94**
**Total**	**5,714**	**6.35**	**5,829**	**6.22**	**6,003**	**6.14**	**6,580**	**6.46**	**7,026**	**6.58**	**31,152**	**6.35**
American Indian/Alaska Native (NH)												
Large metropolitan	189	10.63	178	9.52	189	10.03	211	11.27	273	14.00	**1,040**	**11.17**
Medium/small metropolitan	233	12.30	311	15.62	323	15.52	350	16.25	419	19.60	**1,636**	**16.00**
Nonmetropolitan/rural	505	20.31	643	24.84	655	24.70	762	28.23	792	29.07	**3,357**	**25.47**
**Total**	**927**	**14.95**	**1,132**	**17.48**	**1,167**	**17.60**	**1,323**	**19.59**	**1,484**	**21.79**	**6,033**	**18.37**
Asian/Pacific Islander (NH)												
Large metropolitan	1,415	6.06	1,695	6.33	1,979	6.58	2,275	6.72	2,537	6.70	**9,901**	**6.48**
Medium/small metropolitan	449	7.17	456	6.46	544	7.06	705	8.27	794	8.35	**2,948**	**7.54**
Nonmetropolitan/rural	109	8.25	108	7.48	142	9.00	170	9.56	183	9.35	**712**	**8.78**
**Total**	**1,973**	**6.34**	**2,259**	**6.39**	**2,665**	**6.75**	**3,150**	**7.12**	**3,514**	**7.09**	**13,561**	**6.76**
Hispanic												
Large metropolitan	3,755	6.37	4,098	6.08	4,520	6.19	5,117	6.27	5,690	6.37	**23,180**	**6.25**
Medium/small metropolitan	1,443	6.67	1,810	7.19	2,128	7.56	2,251	7.16	2,722	7.97	**10,354**	**7.37**
Nonmetropolitan/rural	607	9.20	658	8.45	732	8.85	844	9.28	999	10.21	**3,840**	**9.26**
**Total**	**5,805**	**6.65**	**6,566**	**6.52**	**7,380**	**6.72**	**8,212**	**6.71**	**9,411**	**7.05**	**37,374**	**6.75**
**Age group (yrs)**												
10‒14												
Large metropolitan	359	1.05	349	1.01	310	0.91	392	1.16	540	1.59	**1,950**	**1.14**
Medium/small metropolitan	245	1.33	260	1.40	206	1.13	280	1.53	419	2.29	**1,410**	**1.53**
Nonmetropolitan/rural	170	1.57	157	1.50	137	1.37	181	1.83	259	2.69	**904**	**1.78**
**Total**	**774**	**1.22**	**766**	**1.20**	**653**	**1.05**	**853**	**1.37**	**1,218**	**1.97**	**4,264**	**1.36**
15‒24												
Large metropolitan	5,524	8.73	5,809	8.78	5,848	8.53	6,713	9.63	7,079	10.12	**30,973**	**9.17**
Medium/small metropolitan	3,780	9.87	4,048	10.12	4,151	10.18	4,604	11.17	5,103	12.28	**21,686**	**10.74**
Nonmetropolitan/rural	2,620	12.57	2,818	13.35	2,776	13.36	2,945	14.37	3,224	15.82	**14,383**	**13.88**
**Total**	**11,924**	**9.74**	**12,675**	**9.96**	**12,775**	**9.82**	**14,262**	**10.85**	**15,406**	**11.68**	**67,042**	**10.42**
25‒34												
Large metropolitan	7,511	11.00	7,182	10.58	7,631	11.06	8,449	11.78	9,375	12.40	**40,148**	**11.39**
Medium/small metropolitan	4,623	14.15	4,796	14.49	5,142	14.96	5,870	16.39	6,517	17.53	**26,948**	**15.57**
Nonmetropolitan/rural	2,991	17.49	3,038	17.92	3,091	17.89	3,683	20.85	3,914	21.96	**16,717**	**19.25**
**Total**	**15,125**	**12.81**	**15,016**	**12.73**	**15,864**	**13.15**	**18,002**	**14.38**	**19,806**	**15.17**	**83,813**	**13.69**
35‒64												
Large metropolitan	23,952	13.29	25,617	13.53	29,625	15.07	31,912	15.78	32,252	15.62	**143,358**	**14.71**
Medium/small metropolitan	15,267	15.93	16,992	16.86	19,080	18.22	20,775	19.52	22,214	20.85	**94,328**	**18.34**
Nonmetropolitan/rural	10,161	17.60	11,110	18.59	12,213	19.97	13,323	21.86	13,929	23.43	**60,736**	**20.31**
**Total**	**49,380**	**14.79**	**53,719**	**15.36**	**60,918**	**16.81**	**66,010**	**17.86**	**68,395**	**18.36**	**298,422**	**16.69**
≥65												
Large metropolitan	7,222	13.76	6,977	12.92	7,500	13.16	8,386	13.62	9,997	14.49	**40,082**	**13.63**
Medium/small metropolitan	5,021	15.68	4,999	15.05	5,313	15.04	6,010	15.83	7,330	17.34	**28,673**	**15.86**
Nonmetropolitan/rural	3,890	17.55	3,883	17.04	4,168	17.43	4,494	17.78	5,384	19.66	**21,819**	**17.95**
**Total**	**16,133**	**15.12**	**15,859**	**14.42**	**16,981**	**14.61**	**18,890**	**15.14**	**22,711**	**16.38**	**90,574**	**15.19**
**Mechanism of death**												
Firearms												
Large metropolitan	21,468	5.43	20,671	5.03	22,638	5.27	24,886	5.55	26,220	5.60	**115,883**	**5.39**
Medium/small metropolitan	16,246	7.48	16,555	7.29	17,607	7.43	19,722	8.04	21,854	8.62	**91,984**	**7.80**
Nonmetropolitan/rural	13,014	9.99	13,272	9.92	13,930	10.15	15,236	10.98	16,248	11.53	**71,700**	**10.53**
**Total**	**50,728**	**6.81**	**50,498**	**6.52**	**54,175**	**6.72**	**59,844**	**7.16**	**64,322**	**7.43**	**279,567**	**6.95**
Hanging/suffocation												
Large metropolitan	10,187	2.53	11,448	2.76	13,383	3.13	15,140	3.43	16,731	3.66	**66,889**	**3.12**
Medium/small metropolitan	5,728	2.66	6,829	3.08	7,913	3.47	9,050	3.88	10,455	4.41	**39,975**	**3.52**
Nonmetropolitan/rural	3,255	2.67	3,698	3.02	4,342	3.54	5,192	4.22	5,961	4.86	**22,448**	**3.66**
**Total**	**19,170**	**2.58**	**21,975**	**2.88**	**25,638**	**3.28**	**29,382**	**3.66**	**33,147**	**4.04**	**129,312**	**3.31**
Drug poisoning												
Large metropolitan	5,956	1.48	6,602	1.58	7,710	1.77	8,373	1.84	8,216	1.74	**36,857**	**1.70**
Medium/small metropolitan	3,492	1.63	4,138	1.85	4,692	2.01	5,040	2.07	5,125	2.05	**22,487**	**1.94**
Nonmetropolitan/rural	1,843	1.49	2,242	1.77	2,383	1.82	2,566	1.93	2,637	1.96	**11,671**	**1.80**
**Total**	**11,291**	**1.52**	**12,982**	**1.68**	**14,785**	**1.84**	**15,979**	**1.91**	**15,978**	**1.85**	**71,015**	**1.77**
Nondrug poisoning												
Large metropolitan	2,327	0.58	2,315	0.56	2,115	0.49	1,900	0.42	2,105	0.45	**10,762**	**0.50**
Medium/small metropolitan	1,593	0.74	1,522	0.68	1,471	0.63	1,308	0.54	1,397	0.57	**7,291**	**0.63**
Nonmetropolitan/rural	868	0.69	781	0.60	763	0.57	608	0.44	667	0.49	**3,687**	**0.56**
**Total**	**4,788**	**0.64**	**4,618**	**0.60**	**4,349**	**0.54**	**3,816**	**0.46**	**4,169**	**0.49**	**21,740**	**0.55**
Other												
Large metropolitan	4,630	1.16	4,898	1.18	5,068	1.17	5,553	1.24	5,971	1.28	**26,120**	**1.21**
Medium/small metropolitan	1,877	0.87	2,051	0.91	2,209	0.94	2,419	1.00	2,752	1.12	**11,308**	**0.97**
Nonmetropolitan/rural	852	0.67	1,013	0.78	967	0.72	1,024	0.77	1,197	0.90	**5,053**	**0.77**
** Total**	**7,359**	**0.99**	**7,962**	**1.03**	**8,244**	**1.03**	**8,996**	**1.09**	**9,920**	**1.17**	**42,481**	**1.07**

**Abbreviation:** NH = non-Hispanic.

* Rates are age adjusted using the 2000 U.S. standard population, except for the age-specific crude rates. All rates are per 100,000 population.

^†^ By 2006 urbanization classification (https://wonder.cdc.gov/controller/saved/D132/D9F611). Subnational population figures for regions, divisions, states, and counties for 1999 are from the 1990‒1999 series of bridged-race intercensal estimates of the July 1 resident population. Those for 2000 and 2010 are bridged-race April 1 census counts. Those for 2001‒2009 are from the revised 2000‒2009 series of bridged-race intercensal estimates of the July 1 population. Those for 2011‒2015 are bridged-race postcensal estimates of the July 1 resident population from the Vintage 2015 series released by the National Center for Health Statistics on June 28, 2016.

**FIGURE 2:**
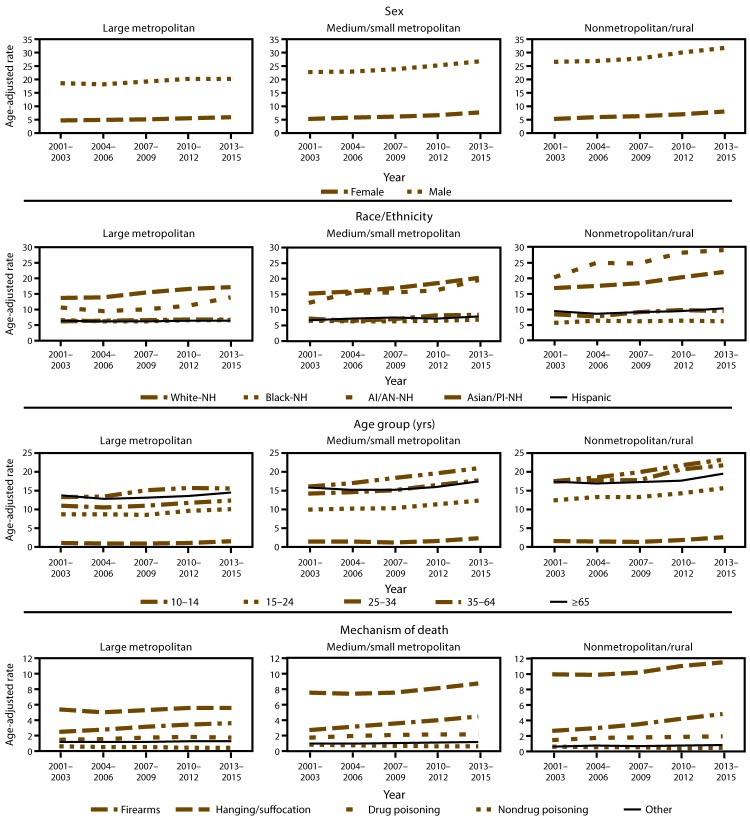
Suicide rates* for selected characteristics among persons aged ≥10 years, by county urbanization level — United States, 2001–2015 **Abbreviations:** AI/AN = American Indian/Alaska Native; NH = non-Hispanic; PI = Pacific Islander. * Per 100,000 residents aged ≥10 years, age adjusted to the 2000 U.S. standard population.

Analysis according to sex, race/ethnicity, age group, and mechanism of death by urbanization level indicated gradual increases in suicide rates occurring across the three urbanization levels for both males and females, with lower rates in large metropolitan counties and higher rates in nonmetropolitan/rural counties (Figure 2). The greatest rate increases occurred in medium/small metropolitan and nonmetropolitan/rural counties for both sexes. Across all three urbanization levels, suicide rates for males were four to five times higher than for females during the study period. By race/ethnicity, rates typically increased in all counties across the study period for all racial/ethnic groups, with greater increases in the medium/small metropolitan and nonmetropolitan/rural counties. Non-Hispanic whites and non-Hispanic American Indian/Alaska Natives had the highest suicide rates across all three urbanization levels, with both groups experiencing greater increases compared with non-Hispanic blacks, non-Hispanic Asian/Pacific Islanders, and Hispanics across the study period. With the exception of non-Hispanic blacks, suicide rates were higher in nonmetropolitan/rural counties. During 2001–2015, rates for all age groups typically increased, with greater increases occurring in medium/small metropolitan and nonmetropolitan/rural counties. For all urbanization levels, higher rates were observed among persons aged ≥25 years, with the highest rates among those aged 35–64 years. Across all urbanization levels, firearms were the most often used mechanism of death, with rates in nonmetropolitan/rural counties almost double those in large metropolitan and medium/small metropolitan counties. Greater increases in rates of suicide by firearms and hanging/suffocation were observed across all urbanization levels whereas lesser increases in rates of suicide by drug poisoning, nondrug poisoning, and other mechanisms were observed during the study period.

## Discussion

During 2001–2015, age-adjusted suicide rates for nonmetropolitan/rural counties were consistently higher than for medium/small and large metropolitan counties. Although other studies have documented these differences (*10*), this report examined annual changes in rates by urbanization level along with trends among and within urbanization levels by sex, race/ethnicity, age group, and mechanism of death. A closer look at annual rate changes revealed substantial increases after 2005 for large metropolitan counties, after 2008 for medium/small metropolitan counties, and after 2007 for nonmetropolitan/rural counties. Although the Great Recession officially began in 2007 and ended in 2009 (*14*), differential effects were observed at different points in different geographic areas (*14*). Economic indicators (e.g., housing foreclosures, poverty, and unemployment) vary by urbanization level, with rural areas usually having greater prevalence of these negative factors (*15*). Factors such as housing foreclosures and overall business cycles negatively affect suicide rates and other health outcomes (*16*–*18*). A combination of these factors likely contributed to the differences in annual suicide rate changes by urbanization level observed in this study. In addition, because U.S. suicide rates were increasing before the Great Recession, other contributors to the changes in rates were likely (*19*).

The differences observed in suicide rates by sex within urbanization levels are consistent with previous findings that age-adjusted suicide rates tend to be higher for men than for women (*3*). This difference is maintained regardless of urbanization level, with differences most notable in nonmetropolitan/rural counties. By race/ethnicity, age-adjusted suicide rates for non-Hispanic American Indian/Alaska Natives are consistently the highest, followed by rates for non-Hispanic whites across all periods; however, when comparing rates by race/ethnicity across urbanization levels, suicide rates are highest for non-Hispanic whites in metropolitan counties and for non-Hispanic American Indian/Alaska Natives in nonmetropolitan/rural counties.

CDC released a technical package of policies, programs, and practices to prevent suicide that includes examples of programs that can be tailored to fit the cultural needs of different racial/ethnic groups (*20*). The technical package is a compilation of a core set of strategies, developed using the best available evidence, that have the greatest prevention potential. For example, a suicide prevention program called Sources of Strength was developed with rural and tribal communities in North Dakota to promote connectedness between youth and adults. The program strategy is to understand and respond to underlying causes of suicidal behavior and promote protective factors against suicidal behavior to prevent adverse outcomes. Sources of Strength is a universal (i.e., programs administered to all children in classrooms regardless of individual risk status) school-based approach to suicide prevention that is designed to build socioecological protective influences across the student population. Youth opinion leaders are recruited from diverse social backgrounds, including some who are at risk for suicidal behavior. They are trained to change the norms and behaviors of their peers by conducting well-defined messaging activities with adult mentoring (*21*). Local implementers might need to tailor this and other programs discussed in CDC’s technical package for suicide prevention to specific cultural practices and traditions of tribes in rural areas.

Another notable finding regarding racial/ethnic differences by urbanization level was identified from analysis of the total age-adjusted rates by urbanization level across the study period. For non-Hispanic blacks, suicide rates do not follow the historical trend of being highest in rural areas. Except during 2004–2006, rates for non-Hispanic blacks in rural areas were consistently lower than rates for non-Hispanic blacks in urban areas, with fluctuations across the entire period. A previous study using data from the 1993 National Mortality Followback Survey to identify risk and protective factors specific to suicide among blacks identified rural residence as a protective factor (*22*). A hypothesis that has been proposed to support this finding is that blacks living in urban areas will be more at risk for suicide due to the stressors and strains of urban life, including unaccustomed social isolation, as well as difficulty acculturating to middle-class suburban living (*22*). 

Findings by age group among urbanization levels revealed increases in rates for all age groups, with the highest rates and greatest rate increases in more rural areas. Within all urbanization levels, the highest rates were observed among persons aged 35–64 years. This age group has been of particular interest given increases in suicide rates among middle-aged whites (*23*). Findings are consistent with those of studies that have identified a pattern of increasing mortality among non-Hispanic white populations, which is in part attributed to increases in drug overdoses, suicides, and alcohol-related mortality, especially among persons aged 45–54 years (*24*). By mechanism of death within each urbanization level, firearms were the most common, with the highest rates and greatest rate increases in rural areas. Among urbanization levels, rates for firearms as the mechanism of death in nonmetropolitan/rural counties were approximately two times the rates of those in metropolitan counties. This might be attributed, in part, to firearm ownership being more common in rural areas and a large number of rural community residents being familiar with firearm use (*25*).

Suicide rates by sex, race/ethnicity, age group, and mechanism of death for the general population are higher in rural communities than in urban areas. In addition to considering differences by sex, race/ethnicity, age group, and mechanism of death, this study underscores the need for analyses both among and within urbanization levels with the goal of designing and implementing tailored suicide prevention efforts. To address suicide in rural areas, the Health Resources and Services Administration has developed activities including epidemiologic studies, research, and programs for primary health care providers. Ongoing work by CDC in suicide prevention from a public health perspective, such as programs that focus on middle-aged men, a group experiencing one of the greatest increases in suicide rates (*3*), is an important step in decreasing overall suicide rates in the United States. Prevention practitioners could use these findings to prioritize and allocate resources for their rural populations as part of efforts to meet the *Healthy People 2020* goal to reduce the suicide rate by 10% (*4*).

## Limitations

The findings in this report are subject to at least five limitations. First, mortality data from the compressed mortality data file missing information on Hispanic origin were excluded when calculating death rates for this group (i.e., no corresponding population denominator data are available). The small fraction (1,970 [0.0036%]) of suicide data excluded might have resulted in a slight underestimation of some rates. Second, use of the 2006 six-level classification scheme does not reflect a number of county reclassifications made in the updated 2013 classification scheme. Although these changes are not reflected in this report, fewer than 10% of counties had different category assignments in the two schemes, with the majority of counties shifted to a more urban category. The effects of using the earlier classification scheme are expected to be minimal. Third, the 2006 National Center for Health Statistics six-level county classifications were further collapsed into three groups (large metropolitan, medium/small metropolitan, and nonmetropolitan/rural) for comparative purposes. Collapsing these categories from six into three might further mask the heterogeneity of certain counties. Fourth, on April 3, 2017, CDC released a revised data file for 2014 to include corrections affecting 125 deaths previously coded to accidental discharge of firearms (ICD-10 codes W32–W34). Of the 125 deaths, 53 were reclassified as intentional self-harm (suicide) by discharge of firearms (ICD-10 codes X72–X74). These revisions were not available for inclusion in this report; however, substantial differences in rates and substantial effects on the results and conclusions in this report are not anticipated. Finally, undercounting or underreporting could have an impact on the findings. Suicides are often undercounted on death certificates and studies have indicated they are differentially undercounted for females and racial/ethnic minorities; therefore, the suicide rates in this report are likely to be underestimates (*26*). The degree of underreporting might also vary by level of urbanization and mechanism of death, and misclassification of race/ethnicity on death certificates, particularly among the American Indian/Alaska Native population, could also affect the findings.

## Future Directions

Detailed analysis of suicide rates among and within urbanization levels by sex, race/ethnicity, age group, and mechanism of death can provide more comprehensive knowledge of national trends and highlight the greater needs in rural communities. Although CDC’s technical package to prevent suicide might help states and communities focus on strategies supported by the best available evidence, ongoing evaluation of suicide prevention programs is still necessary to advance suicide prevention efforts (*20*). This study emphasizes the need for development and evaluation of suicide prevention efforts specific to rural communities. Innovative prevention strategies, such as telebehavioral health (i.e., telephone-, video-, and web-based technologies), are a promising option to increasing access to health care and mental health care in rural communities (*20*); however, rural communities often have limited access to the Internet (*27*). Future analyses might target identification of rural communities whose populations are at greatest risk for suicide to prioritize Federal Communications Commission broadband access needs, furthering telebehavioral health approaches and helping to reduce suicide rates.

In the move toward more comprehensive suicide prevention approaches, prevention specialists might need to consider tailoring specific programs to the needs of various racial/ethnic groups for effective prevention efforts. Although all-cause mortality rates for blacks are higher than those for whites, suicide is the only leading cause of death for which the death rate is lower among blacks compared with whites for all age groups (*28*). Among American Indian/Alaska Natives, suicide rates in rural areas are substantially higher than the already high rates for this population. Future studies should attempt to identify risk factors and protective factors for suicide specific to racial/ethnic groups.

## Conclusion

Nonmetropolitan/rural counties have higher suicide rates than medium/small and large metropolitan counties. Over the study period, notable changes were observed in rates by urbanization level. A combination of factors, including economic and health indicators, likely contributed to the differences in annual suicide rate changes by urbanization level (*14**–**18*) observed in this study. Trends in suicide rates by sex, race/ethnicity, age group, and mechanism of death that are observed in the general population indicate that rates are consistently higher in rural communities. Findings from this study underscore the need to identify protective factors as part of comprehensive suicide prevention efforts, particularly in rural areas. Additional information on suicide rates in rural areas might be used to evaluate current suicide prevention efforts and determine which rural communities to prioritize when allocating public health and mental health resources.
